# FACIT-fatigue score and treatment quality assessment for paroxysmal nocturnal hemoglobinuria patients treated with Eculizumab in Türkiye

**DOI:** 10.1371/journal.pone.0355720

**Published:** 2026-09-11

**Authors:** Zehra Narlı Özdemir, Fatma Keklik Karadağ, Nuray Gül Acar, Merve Aydoğan, Filiz Yavaşoğlu, Fatma Temiz, Ajda Güneş, Gülşah Akyol, Haşim Atakan Erol, Özgür Mehtap, Vahap Okan, Merve Kakçı, İsmail Can Kendir, Turhan Köksal, Utku Iltar, Ramazan Esen, Gülten Korkmaz, Mesut Ayer, Esin Oğuz, Ozan Salim, Anıl Tombak, Gülsüm Özet, Mehmet Sönmez, Nil Güler, Mehmet Yilmaz, Mehmet Ali Özcan, Ali Ünal, Tülin Tuğlular, Selami Koçak Toprak, Birol Güvenç, Mustafa Nuri Yenerel, Osman İlhan, Güray Saydam, Fahri Şahin

**Affiliations:** 1 Department of Hematology, School of Medicine, TOBB Economics and Technology University, Ankara, Türkiye; 2 Department of Hematology, School of Medicine, Ege University, İzmir, Türkiye; 3 Department of Hematology, School of Medicine, Çukurova University, Adana, Türkiye; 4 Department of Hematology, School of Medicine, Ankara University, Ankara, Türkiye; 5 Department of Hematology, School of Medicine, Eskişehir Osmangazi University, Eskişehir, Türkiye; 6 Department of Hematology, School of Medicine, Marmara University, İstanbul, Türkiye; 7 Department of Hematology, School of Medicine, Erciyes University, Kayseri, Türkiye; 8 Department of Hematology, School of Medicine, Kocaeli University, İzmit, Türkiye; 9 Department of Hematology, School of Medicine, Gaziantep University, Gaziantep, Türkiye; 10 Department of Hematology, School of Medicine, Dokuz Eylül University, İzmir, Türkiye; 11 Department of Hematology, School of Medicine, Pamukkale University, Denizli, Türkiye; 12 Department of Hematology, School of Medicine, Karadeniz Teknik University, Trabzon, Türkiye; 13 Department of Hematology, School of Medicine, Akdeniz University, Antalya, Türkiye; 14 Department of Hematology, School of Medicine, Van Yüzüncü Yıl University, Van, Türkiye; 15 Department of Hematology, Bilkent City Hospital, Ankara, Türkiye; 16 Department of Hematology, Çam and Sakura City Hospital, İstanbul, Türkiye; 17 Department of Hematology, School of Medicine, Mersin University, Mersin, Türkiye; 18 Department of Hematology, School of Medicine, Sanko University, Gaziantep, Türkiye; 19 Department of Hematology, Çapa School of Medicine, İstanbul University, İstanbul, Türkiye; 20 Department of Hematology, School of Medicine, Lokman Hekim University, Ankara, Türkiye; Tekirdag Namik Kemal University: Tekirdag Namik Kemal Universitesi, TÜRKIYE

## Abstract

**Objective:**

Paroxysmal nocturnal hemoglobinuria (PNH) is an ultra-rare acquired clonal abnormality that makes hematopoietic cells highly vulnerable to complement-mediated destruction. Fatigue is one of the most commonly reported unresolved symptoms by patients with PNH. In this study, the fatigue status of PNH patients receiving eculizumab treatment and the factors affecting fatigue were evaluated using a quality-of-life questionnaire.

**Materials and Methods:**

The study included 18 centers from Türkiye. The quality of life of PNH patients receiving eculizumab treatment was assessed through face-to-face surveys using the Functional Assessment of Chronic Illness Therapy-Fatigue (FACIT-Fatigue) scale.

**Results:**

A total of 104 PNH patients treated with eculizumab were included in the study, of whom 51 (49%) were female and 53 (51%) were male. The mean (±SD) age of the patients was 48.04 (±16.16) years at the time of the survey. The mean (±SD) FACIT-Fatigue score was 32.63 (±11.79). A total of 39 (37.5%) patients had a fatigue score of 30 or below, which was defined as severe fatigue. Patients with severe fatigue had lower hemoglobin levels both at diagnosis and at the time of the survey [8.05 g/dL vs. 9.15 g/dL (p = 0.017) and 10.09 g/dL vs. 11.38 g/dL (p = 0.006), respectively]. The mean (±SD) duration of additional medication use was longer in the severe fatigue group compared to patients with a fatigue score >30 [97.92(±51.98) months vs. 75.94 (±50.55) months, p = 0.039]. In the multivariate analysis, the independent variables ‘female gender,’ ‘presence of comorbidities,’ ‘duration of additional medication,’ and ‘initial hemoglobin level’ were statistically significantly associated with severe fatigue. The female gender increased the likelihood of severe fatigue by 3.3 times, while the presence of comorbidities increased it by 7.1 times.

**Conclusion:**

In this study, which evaluated the fatigue status of PNH patients at various stages of eculizumab treatment in a real-life setting, more than one-third of the patients reported severe fatigue. Female patients experienced severe fatigue more than male patients, and anemia was not the sole factor contributing to severe fatigue in PNH patients. Comorbidities and medications related to these conditions also played a significant role in their quality of life.

## Introduction

Paroxysmal nocturnal hemoglobinuria (PNH) is a rare, acquired, life-threatening, clonal hematopoietic stem cell disorder with a global prevalence range between 10 and 20 cases per million [1,2]. PNH is characterized by chronic intravascular hemolysis caused by uncontrolled terminal complement activation. PNH can affect any age group but is most diagnosed in the third decade of life. There is no clear geographic, ethnic, or sex preference. Its manifestations vary and include fatigue, dyspnea, abdominal pain, hemoglobinuria, and smooth muscle dystonia (e.g., esophageal spasms and erectile dysfunction [3].

Fatigue is the most frequently reported symptom in PNH, affecting 80% of patients. It is often severe [4] and has a substantial impact on quality of life (QoL) [5]. However, fatigue can be difficult to quantify and is sometimes overlooked due to time constraints. Additionally, patients may struggle to describe their experiences in detail due to their variable nature. To address this challenge, clinicians have turned to questionnaires and scales to assess the severity of fatigue in PNH patients more objectively. One notable example is the FACIT-Fatigue questionnaire, which is highly regarded for its ability to systematically analyze different aspects of fatigue, allowing clinicians to gain a more comprehensive understanding of its impact on quality of life [6].

The Functional Assessment of Chronic Illness Therapy-Fatigue (FACIT-Fatigue) scale was originally developed to more accurately assess fatigue related to anemia in cancer patients. It was later adapted for evaluating fatigue in other diseases, including PNH [7]. This instrument has been validated in PNH patients and is widely used in clinical trials and the International PNH Registry [8]. The FACIT-Fatigue scale for PNH consists of 13 items, each scored from 0 to 4, with a maximum total score of 52. Higher scores indicate less fatigue**.**

Eculizumab is a humanized monoclonal antibody that targets C5, a protein of the terminal complement system, and has been approved for the treatment of patients with PNH since 2006 [9]. Several studies have demonstrated that eculizumab significantly reduces fatigue, as indicated by improved FACIT-Fatigue scores [9–12].

The treatment of PNH has been revolutionized by the introduction of the anti-C5 agent eculizumab; however, eculizumab is not a curative treatment for PNH, and there is still unmet need including residual anemia during eculizumab treatment. Indeed, the hematological benefit obtained during eculizumab treatment in PNH patients is highly heterogeneous. More than one- third of patients do not achieve complete normalization of hemoglobin, while the remaining patients continue to experience some degree of anemia, in some cases requiring regular red blood cell transfusions [13].

Eculizumab was the only available complement inhibitor therapy in Türkiye. For more than 10 years, eculizumab has been used in the treatment of PNH patients in our country; however, access to innovative therapies remains limited to clinical trials. Fatigue is one of the most common unresolved symptoms reported by patients with PNH. Therefore, assessing how patients feel and determining their fatigue levels under ongoing eculizumab treatment has gained importance. In this study, the fatigue status of PNH patients receiving eculizumab treatment and the factors affecting fatigue were evaluated using a FACIT-Fatigue scale.

## Methods

### Study design, setting, and data collection

Eighteen centers from Türkiye participated in the study. Patients aged ≥18 years with a confirmed diagnosis of PNH who had been treated with eculizumab were included. All diagnostic procedures were performed using flow cytometry to assess GPI-linked antigens on red blood cells and neutrophils.

Demographic data of the patients, including age, sex, age at diagnosis, eculizumab dose and dosing interval, levels of hemoglobin and lactate dehydrogenase (LDH) and transfusion requirement and number of transfused erythrocyte units were collected from hospital medical records.

The quality of life of PNH patients receiving eculizumab treatment was assessed using the FACIT-Fatigue scale through face- to-face surveys. Patients are asked to complete patient-assessment questionnaires, including the FACIT-Fatigue scale, PNH-related complications [pulmonary hypertension (PHT), chronic renal failure, thrombosis, and others], comorbidities, medications regularly used other than PNH, and the travel time from their place of residence to the treatment center. All surveys were conducted between 01/01/2024 and 31/12/2024. Patients unable to complete patient-reported assessment questionnaires due to neuropsychiatric disorders or concomitant use of tranquilizers and sedatives were excluded. Beyond these exclusions, concomitant medications were systematically recorded and categorized by class, including antihypertensives, oral antidiabetics, and lipid-lowering agents. It is noteworthy that all patients with non-PNH comorbidities were actively receiving chronic treatment for their underlying conditions at the time of assessment. Rather than analyzing the individual pharmacological effects of each drug, our analysis focused on the presence of these comorbidities and the duration of chronic medication use as potential clinical confounders. This allowed us to assess whether the cumulative burden of chronic disease management independently influenced the subjective experience of fatigue.

FACIT- Fatigue is assessed using a 13-item self-administered questionnaire **(Supplementary file)**, with each question offering five ordinal response options (’not at all,’ ‘a little bit,’ ‘somewhat,’ ‘quite a bit,’ and ‘very much’) [14]. Each item contributes equally (score range: 0–4) to a total score ranging from 0 to 52, where higher scores indicate less fatigue [15]. A score will only be calculated if at least 7 of the 13 questions have a response. In our study, all the questions had responses. A score of less than 30 indicates severe fatigue [6]. While clinical assessments focused on PNH-related fatigue, objective measures for sleep disorders (e.g., Pittsburgh Sleep Quality Index) and depression scales were not formally administered due to the study's primary focus on the FACIT-Fatigue tool in a routine clinical setting. This is addressed in the limitations section.

Ethical approval was obtained from the Ege University Medical Research Ethics Committee, with approval number 23-11.1T/34. Written informed consent was obtained from each participant without any obligation to complete the study if they did not want to.

### Statistical method

The demographic characteristics of the patients and the disease-related data were appropriately summarized, and numerical and categorical variables were presented in a table using appropriate methods (minimum, maximum, mean, standard deviation, frequency, and percentage). Continuous variables were summarized, while categorical variables were analyzed using cross-tables. Cross tables (Chi-square test) were used for categorical variables. The Student's t-test was used for related samples. The strength and direction of the monotonic relationship between variables were assessed using the Pearson Correlation analysis. Logistic regression analysis was used to model the relationship between a dependent variable and one or more independent variables when the dependent variable was categorical. Before multivariable logistic regression analysis, multicollinearity among candidate predictors was evaluated using variance inflation factors (VIFs) and tolerance values. No evidence of significant multicollinearity was detected (all VIFs < 1.2 and all tolerance values > 0.8). Missing data was handled using complete-case analysis. Only participants with complete data for all variables included in each specific analysis were analyzed. No imputation of missing values was performed. Accordingly, the multivariable logistic regression analysis included 86 participants.

All statistical analyses were performed using the SPSS software package (SPSS 21, Inc., Chicago, IL).

## Results

One hundred four patients with PNH treated with eculizumab were included in the study, of whom 51 (49%) were female and 53 (51%) were male. The mean (±SD) age of the patients was 48.04 (±16.16) years at the time of the survey. Among the patients, 85 (81.7%) received 900 mg of eculizumab, while 16 (15.4%) received 1200 mg. Regarding dosing intervals, 97 patients (93.3%) received eculizumab every 14 days, while 5 patients (4.8%) received it every 12 days. Thrombosis was observed in 24 patients (23.1%), PHT in 4 patients (3.8%), renal failure in 2 patients (1.9%), and other complications in 15 patients (14.4%), while no PNH-related complications were observed in 59 patients (56.7%).

A total of 41 patients (39.4%) were receiving chronic treatment unrelated to PNH due to at least one chronic disease, with the most common conditions being diabetes (9 patients, 8.7%), coronary artery disease (4 patients, 3.8%), and hypertension (3 patients, 2.9%).

Regarding travel time to the treatment center, 50 patients (48.1%) had a travel time of less than 1 hour, while 54 patients (51.9%) had a travel time of more than 1 hour. The demographic features of the patients are presented in Table 1.

**Table 1:** Demographic features of the patients.

	Frequency (%) (n = 104)
**Sex**	
**•** Female	51 (49%)
**•** Male	53 (51%)
**Eculizumab dose (mg)**	
**•** 900	85 (81.7%)
**•** 1200	16 (15.4%)
**•** Others	3 (2.9%)
**Eculizumab interval (every [X] day)**	
**•** 12	5 (4.8%)
**•** 13	0 (0%)
**•** 14	97 (93.3%)
**•** Others	2 (1.9%)
**Complications associated with PNH**	
**•** None	59 (56.7%)
**•** Thrombosis	24 (23.1%)
**•** PHT	4 (3.8%)
**•** Renal failure	2 (1.9%)
**•** Others	15 (14.4%)
**The travel time between the medical center and the patient’s residence**	
**•** ≤ 1 hour	50 (48.1%)
**•** > 1 hour	54 (51.9%)
**Comorbidities not associated with PNH**	
**•** None	63 (60.6%)
**•** HT	3 (2.9%)
**•** DM	9 (8.7%)
**•** CAD	4 (3.8%)
**•** CKD	2 (1.9%)
**•** NPD	3 (2.9%)
∙ MDS	1 (1.0%)
**•** Others	19 (18.3%)
**Medications not associated with PNH**	
**•** Yes	41 (39.4%)
**•** No	63 (60.6%)
**Total FACIT-Fatigue score**	
**•** ≤ 30 points	39 (37.5%)
**•** >30 points	65 (62.5%)

Data is presented as frequency (%).

CAD, coronary artery disease; CKD, chronic kidney disease; DM, diabetes mellitus; HT, hypertension; max, maximum; MDS, myelodysplastic syndrome; min, minimum; NPD, neuropsychiatric disease; PHT, pulmonary hypertension; PNH, paroxysmal nocturnal hemoglobinuria.

The mean LDH level at the time of diagnosis was 1210.75 IU/L (SD: 1014.07, range: 150–7129 IU/L), while at the time of the survey, it was 313.13 IU/L (SD: 263.95, range: 148–1857 IU/L).

The mean change in LDH level was 902.02 IU/L (SD: 1072.77, range: −1056–6882 IU/L). The mean hemoglobin level at diagnosis was 8.71 g/dL (SD: 2.13, range: 3.4–14.1 g/dL), and at the time of the survey, it was 10.9 g/dL (SD: 2.34, range: 6.6–15.7 g/dL). The mean change in hemoglobin level was 2.26 g/dL (SD: 2.70, range: −4.5 to 11.4 g/dL). Twenty-five patients (24%) still required transfusions during the last year of the survey period. The median number of erythrocyte transfusion units was 3 per year (range, 1–12). One patient received oral iron supplementation before initiation of eculizumab but did not require further iron supplementation after eculizumab treatment. In Türkiye, erythropoiesis‑stimulating agents (ESAs) are not approved for the treatment of anemia in patients with PNH; consequently, none of the patients received ESAs for anemia that remained unresolved after eculizumab therapy. The mean duration of eculizumab therapy was 85.44 months (SD: 52.65, range: 10.3–181.73 months). The remaining demographic and clinical characteristics of the patients are presented in Table 2.

**Table 2:** Demographic and clinical characteristics of patients.

Characteristics	Minimum	Maximum	Mean (SD)
Age at survey time (year)	20	87	48.04 (16.16)
Age at diagnosis time (year)	4	79	39.46 (16.68)
LDH at diagnosis time (IU/L)	150	7129	1210.75 (1014.07)
Hb at diagnosis time (gr/dl)	3.4	14.1	8.71 (2.13)
LDH at survey time (IU/L)	148	1857	313.13 (263.95)
Hb at survey time (gr/dl)	6.6	15.7	10.90 (2.34)
LDH change (first LDH)- (last LDH)	−1056.00	6882.00	902.02 (1072.77)
Hb change (last Hb)- (first Hb)	−4.50	11.40	2.26 (2.70)
FACIT-Fatigue Score	6.00	51.00	32.63 (11.79)
Duration of eculizumab exposure (month)	10.3	181.73	85.44 (52.65)
Duration of additional medication (month)	1.00	179.00	83.91 (51.91)

Hb, hemoglobin; LDH, lactate dehydrogenase; SD, standard deviation.

In the FACIT-Fatigue scale, where the maximum score is 52, a score of 30 or below was defined as severe fatigue. The mean (±SD) FACIT-Fatique score was 32.63 (±11.79). A total of 39 patients (37.5%) had a fatigue score of 30 or below. The lowest mean (±SD) score (2.11 ± 1.079) was given to the statement: ‘I feel tired’ (Question 4). The highest mean (±SD) score (3.33 ± 0.886) was given to the statement: ‘I am too tired to eat’ (Question 10). All scores assigned to survey questions are presented in Table 3.

**Table 3:** Scores assigned to survey questions.

Question number	N	Minimum	Maximum	Mean (SD)
Q1	104	0	4	2.14 (1.07)
Q2	104	0	4	2.20 (1.07)
Q3	104	0	4	2.79 (1.24)
Q4	104	0	4	2.11 (1.08)
Q5	104	0	4	2.39 (1.22)
Q6	104	0	4	2.44 (1.34)
Q7	104	0	4	2.32 (1.05)
Q8	104	0	4	2.63 (1.07)
Q9	104	0	4	2.47 (1.15)
Q10	104	1	4	3.33 (0.89)
Q11	104	0	4	2.81 (1.29)
Q12	104	0	4	2.68 (1.32)
Q13	104	0	4	2.38 (1.40)

Q, question.

Severe fatigue was found to be more common in women than in men (49% vs. 26.4%, p = 0.025), in patients with additional diseases other than PNH compared to those without (56.1% vs. 25.4%, p = 0.002), and in patients taking regular medication for comorbid conditions compared to those who were not (52.4% vs. 27.4%, p = 0.013). There was no significant relationship between severe fatigue and age, having LDH levels exceeding 1.5 times or 2 times the upper limit of normal (ULN) at the time of diagnosis, nor with the treatment center distance based on a travel time of more than or less than 1 hour (p > 0.05).

Patients who experienced severe fatigue had lower mean (±SD) hemoglobin levels both at diagnosis and at the time of the survey [8.05 (±2.16) g/dL vs. 9.15 (±2.01) g/dL (p = 0.017) and 10.09 (±2.46) g/dL vs. 11.38 (±2.14) g/dL (p = 0.006), respectively]. The mean (±SD) duration of additional medication use was longer in the severe fatigue group compared to patients with a fatigue score >30 points [97.92 (±51.98) months vs. 75.94 (±50.55) months, p = 0.039]. There was no significant difference in age at diagnosis and at survey time, LDH levels at diagnosis and at survey time, or the mean change in LDH and hemoglobin levels between patients with a fatigue score >30 points and ≤ 30 points (p > 0.05). Factors affecting fatigue score are presented in Table 4.

**Table 4:** Factors affecting fatigue score.

	Fatigue (FACIT-Fatigue Score)	N	Mean (SD)	P value
Age at survey time (year)	≤ 30 points	39	50.69 (16.90)	0.196
	>30 points	65	46.45 (15.61)	
Age at diagnosis time (year)	≤ 30 points	39	41.15 (18.04)	0.423
	>30 points	64	38.42 (15.86)	
LDH at diagnosis time (IU/L)	≤ 30 points	36	1233.17 (990.47)	0.866
	>30 points	55	1196.07 (1038.02)	
Hb at diagnosis time (gr/dl)	≤ 30 points	35	8.05 (2.16	**0.017**
	>30 points	53	9.15 (2.01	
LDH at survey time (IU/L)	≤ 30 points	39	382.67 (369.46)	0.082
	>30 points	65	271.42 (162.36	
Hb at survey time (gr/dl)	≤ 30 points	39	10.09 (2.46	**0.006**
	>30 points	65	11.38 (2.14	
Duration of additional medication (months)	≤ 30 points	37	97.92 (51.98	**0.039**
	>30 points	65	75.94 (50.55	
LDH change (first LDH)- (last LDH)	≤ 30 points	36	880.75 (1087.43	0.879
	>30 points	55	915.95 (1072.90)	
Hb change (last Hb)- (first Hb)	≤ 30 points	35	2.10 (3.35)	0.657
	>30 points	53	2.36 (2.19)	

FACIT-Fatigue Score, Functional Assessment of Chronic Illness Therapy-Fatigue Score; Hb, hemoglobin; LDH, lactate dehydrogenase; SD, standard deviation.

A Pearson correlation analysis revealed a significant moderate positive correlation between final hemoglobin levels and fatigue. (r = .319, n = 104, p = .001). A mild positive correlation was observed between the initial hemoglobin levels and fatigue (r = .317, n = 88, p = .003).

In the multivariable logistic regression model, female sex, presence of comorbidities, duration of additional medication, and hemoglobin level at diagnosis were significantly associated with severe fatigue in patients with PNH. Female sex increased the likelihood of severe fatigue by approximately 3.3-fold (odds ratio [OR] = 3.299; 95% confidence interval [CI], 1.041–10.452; p = 0.042), while the presence of comorbidities increased it by more than 7-fold (OR = 7.098; 95% CI, 2.038–24.717; p = 0.002). A 1-unit increase in the duration of additional medication raised the likelihood of severe fatigue by 2.1% (OR = 1.021; 95% CI, 1.008–1.034; p = 0.001), whereas a 1-unit increase in hemoglobin level at diagnosis reduced it by 28.4% (OR = 0.716; 95% CI, 0.545–0.942; p = 0.017). Interestingly, in the multivariable analysis, hemoglobin level at the time of the survey was not significantly associated with severe fatigue (p = 0.076). The multivariable analysis of parameters contributing to severe fatigue in PNH patients is presented in Table 5.

**Table 5:** Multivariate analysis of parameters contributing to the sense of severe fatigue in PNH Patients.

	P value	Odds ratio	95% Confidence interval	
			Lower	Upper
Female gender	**0.042**	3.299	1.041	10.452
Comorbidities not associated with PNH	**0.002**	7.098	2.038	24.717
Duration of additional medication (month)	**0.001**	1.021	1.008	1.034
Hb at diagnosis time (gr/dl)	**0.017**	0.716	0.545	0.942
Hb at survey time (gr/dl)	0.076	0.789	0.608	1.025
Constant	0.298	5.362		

Hb, hemoglobin; PNH, paroxysmal nocturnal hemoglobinuria.

## Discussion

Previous studies have proven that eculizumab can lead to positive treatment outcomes and sustained benefits for patients with PNH, including improvements in FACIT-Fatigue scores, reductions in LDH levels, increases in hemoglobin concentrations, and transfusion avoidance [16]. However, there are limited studies evaluating the fatigue status of PNH patients at any stage of eculizumab treatment and assessing the factors affecting fatigue. This is particularly relevant given that the subject of quality of life is of great importance, especially for ultrarare diseases that remain incurable today, as most of these diseases are chronic, progressive, and debilitating over time. The lack of hope for a cure has a significant psychological impact, and the internal struggle experienced in the face of a diagnosis without the expectation of permanent relief deserves not only recognition but also clinical investigation.

In our study, with a mean eculizumab exposure of 85.44 (SD 52.65) months, 37.5% of the patients experienced severe fatigue. As previously mentioned, eculizumab is the only available complement inhibitor treatment in Türkiye, and our results highlight an unmet need for patients in the country. Dingli et al. demonstrated that in patients with PNH receiving complement inhibitor treatment, statistically significant associations (p < 0.05) were observed between fatigue severity, health-related quality of life, and functional impairment [17].

Our results showed that female patients experienced more fatigue, and that severe fatigue was associated with the presence of comorbidities, the duration of additional medication use, and low initial hemoglobin levels at diagnosis. Eculizumab is administered via the intravenous route, and patients must visit the hospital every two weeks. In our study, we investigated the effect of the time patients had to travel to access their chronically used medication, eculizumab, on their feeling of fatigue. The travel time to the center had no effect on the presence of severe fatigue.

Similar results from a patient survey in the United States (US) showed that PNH patients receiving eculizumab or ravulizumab therapy demonstrated a significant burden of illness and had scores below the average population norms on the FACIT-Fatigue scale. Additionally, most patients on eculizumab (88.6%; n = 31/35) and ravulizumab (74.7%; n = 65/87) reported fatigue symptoms, and many patients remained anemic with hemoglobin levels ≤ 12 g/dL, despite the fact that most patients had been receiving C5i therapy for ≥ 3 months [18]. In our cohort of 104 PNH patients, the mean hemoglobin level at the time of the survey was 10.9 g/dL (SD: 2.34, range: 6.6–15.7 g/dL), and a 1 g/dL increase in the initial hemoglobin level reduced the feeling of severe fatigue by 28.4%. Although a mild positive correlation was observed between the total fatigue score and both the initial and final hemoglobin levels (p = 0.003 and p = 0.001, respectively), the final hemoglobin level at survey time had no effect on the presence of severe fatigue, as defined by a FACIT-Fatigue score ≤ 30.

In two phase 3 clinical studies and two cross-sectional patient surveys, patients receiving terminal complement inhibitor treatments reported mean FACIT-Fatigue scores of 29–44 [18–21], whereas the mean FACIT-Fatigue score in the general population of the US and Europe was 43 [22,23]. In our study, the mean FACIT-Fatigue score was 32.63, which was consistent with previous reports [18–21] on PNH patients. Our results proved that anemia was not the only factor contributing to severe fatigue in PNH patients; comorbid diseases and medications related to these comorbidities were also highly important for the quality of life of PNH patients.

Our finding that 37.5% of patients report severe fatigue despite long-term eculizumab (mean 85.4 months) underscores the limitations of terminal C5 inhibition. This ongoing burden highlights how rapidly the therapeutic landscape for PNH has evolved in recent years, moving beyond traditional terminal complement inhibition to include novel proximal and next-generation strategies such as pegcetacoplan, iptacopan, crovalimab, and danicopan [24]. In contrast to conventional C5i, emerging data on proximal inhibitors like pegcetacoplan have shown superior control of extravascular hemolysis, leading to mean FACIT-Fatigue improvements of 9 points or more in head-to-head trials [25,26]. This suggests that for patients with persistent fatigue in our cohort, switching to proximal inhibition might address the ‘unmet need’ highlighted by our results.

Although our study identified significant predictors of fatigue through multivariate analysis—such as female sex and comorbidity duration—the sample size limited our ability to perform head-to-head subgroup comparisons between different dosing regimens of eculizumab (900 mg vs 1200 mg). Future multicenter studies with larger cohorts should employ propensity score matching to isolate the impact of treatment intensification on residual fatigue.

### Clinical context and confounders

Fatigue in PNH is a multifactorial symptom influenced not only by disease activity but also by socioeconomic status, sleep quality, psychological well-being, concomitant medications, and residual anemia management. Our study may therefore be subject to the influence of unmeasured confounding factors. In particular, medications such as sedatives, antidepressants, and antihypertensive agents may independently contribute to fatigue perception. Although patients who were unable to complete questionnaires due to neuropsychiatric conditions or the use of sedatives/tranquilizers were excluded, detailed and systematically categorized data on other concomitant medications were not collected. This limitation precluded a comprehensive evaluation of their potential confounding effects.

Previous research has demonstrated that sleep disturbances are closely associated with fatigue across various chronic conditions, including PNH. Specifically, reduced sleep duration, impaired sleep efficiency, and poor sleep quality have all been significantly linked to increased fatigue and diminished functional capacity [27–30]. However, objective assessment of sleep disorders using validated instruments such as the Pittsburgh Sleep Quality Index was not performed in the present study, which may have limited the accurate characterization of sleep-related contributors to fatigue.

In addition, psychological factors such as anxiety and depression are strongly correlated with greater fatigue severity and are known to account for a substantial proportion of fatigue variability in chronic disease populations [27,31,32]. Evidence from real-world data in PNH patients further supports this, showing that the anxiety/depression dimension was significantly worse among untreated patients (p = 0.0334), while both untreated (p = 0.0001) and treated (p = 0.0008) individuals exhibited significantly poorer general health scores compared to controls [30]. These factors may therefore influence patient-reported fatigue outcomes.

Our findings indicated that female patients reported significantly higher levels of fatigue—specifically, female gender increased the likelihood of severe fatigue by 3.3 times—necessitating a deeper examination of sex-specific biological pathways. Biologically, sex hormones are not merely clinical markers but active immunomodulators; for instance, estrogen has been shown to regulate the NLRP3 inflammasome activation [34], a key pathway in the inflammatory cascade that may exacerbate the subjective perception of fatigue in PNH patients. This is further influenced by hormonal fluctuations where luteal progesterone levels correlate with central nervous system-mediated fatigue responses [33]. Additionally, women are predisposed to deteriorated iron metabolism due to life stages such as menstruation or pregnancy, which may synergize with chronic PNH-related hemolysis [35]. This synergy likely impairs mitochondrial energy production and cellular respiration beyond what is expected from complement-mediated hemolysis alone. Furthermore, sex-specific psychosocial dynamics, including higher levels of repetitive negative thinking and stress-induced sleep disturbances, act as independent contributors to fatigue severity [36]. Consequently, the observed fatigue in PNH should be viewed as a multidimensional construct where hormonal, metabolic, and psychological confounders interact. Ultimately, the presence of persistent fatigue despite long-term complement inhibition should encourage clinicians to perform a comprehensive, multidimensional assessment rather than attributing residual fatigue solely to ongoing hemolysis. In clinical practice, when faced with such refractory fatigue, a thorough evaluation encompassing residual anemia management, comorbidities, concomitant medications, sleep disorders, and psychological factors is highly warranted to optimize patient outcomes.

### Study limitations

Our study has several limitations. First, the cross-sectional design of this study inherently precludes drawing causal inferences between fatigue and the associated clinical variables identified in our regression analysis, meaning that longitudinal studies are required to confirm these relationships. Second, fatigue was evaluated using the FACIT-Fatigue scale, a widely validated patient-reported outcome measure; however, as it relies on patients’ perceptions, it may introduce variability and be influenced by subjectivity and recall bias. Third, objective assessments of fatigue, such as actigraphy or biological markers, were not performed. Fourth, sleep disorders were not evaluated using standardized or validated tools (e.g., Pittsburgh Sleep Quality Index), which represents an important limitation; this absence may limit our ability to distinguish between fatigue secondary to sleep pathology versus fatigue directly related to PNH pathophysiology. Fifth, concomitant medications that may influence fatigue—such as sedatives, antidepressants, and antihypertensive agents—were not systematically recorded or analyzed, limiting the ability to assess their potential confounding effects. Sixth, psychological factors such as depression and anxiety were not formally assessed, despite their known impact on fatigue severity. Seventh, socioeconomic status was not evaluated, which may have influenced patient-reported outcomes. Furthermore, regarding our statistical approach, the multivariable logistic regression model included 39 events and 5 covariates, resulting in an events-per-variable (EPV) ratio of 7.8. This is below the conventional recommendation of 10. Although the model identified statistically significant independent predictors, the relatively low EPV may have reduced the stability of the regression estimates and should be considered when interpreting the findings. Finally, subgroup analyses comparing different treatment modalities were not feasible due to the limited sample size, and the generalizability of the findings may be constrained by the geographic setting.

### Future directions

Future research directions should include several key considerations to further refine the management of fatigue in PNH. First, longitudinal studies with time-dependent covariates are warranted to better characterize the trajectory of fatigue over time and to capture potential fluctuations associated with disease course. Such designs, combined with advanced statistical techniques like propensity score matching, would allow for robust head-to-head subgroup comparisons between different therapeutic strategies—specifically comparing terminal C5 inhibition (e.g., eculizumab) versus proximal inhibition (e.g., pegcetacoplan)—thereby allowing for a more robust exploration of causal relationships between treatment intensification and residual fatigue. Second, future analyses should incorporate biomarker-driven subgroups (e.g., LDH, reticulocyte levels) and genetic polymorphisms to support more personalized treatment decisions. Third, to address the current gap in socioeconomic data, future research should systematically collect variables such as educational attainment and household income, utilizing multilevel modeling to explore the complex pathways between these social determinants and fatigue severity. Finally, we emphasize the critical importance of adopting multidimensional fatigue assessment in future studies. Integrating patient-reported outcomes, such as the FACIT-Fatigue scale, with objective and complementary measures—specifically sleep quality indices (e.g., PSQI) and activity-based monitoring (e.g., actigraphy)—will provide a more holistic evaluation. This comprehensive approach, by capturing physical, psychological, and behavioral dimensions, would allow for a more accurate characterization of fatigue and help mitigate the impact of residual confounding in clinical practice.

## Conclusion

Our study, which evaluated the fatigue status of PNH patients at various stages of eculizumab treatment in a real-life setting, demonstrated that PNH patients experience significant fatigue and have an impaired quality of life. More than one-third of the patients reported severe fatigue, with female patients experiencing it more severely than male patients. Anemia was not the sole factor contributing to severe fatigue in PNH patients; comorbid diseases and medications related to these conditions also played a significant role in their quality of life. Given the negative impact of fatigue on patients’ quality of life and functional capacity, the importance of effective PNH management becomes evident. Our findings underscore an unmet need for PNH patients in Türkiye.
